# Supplementary material for: Efficient Lignin Fractionation from Scots Pine (Pinus sylvestris) Using Ammonium-Based Protic Ionic Liquid: Process Optimization and Characterization of Recovered Lignin
Source: Polymers (Basel). 2022 Oct 31;14(21):4637. doi: 10.3390/polym14214637 (PMC9657931; doi:10.3390/polym14214637)
Supplement: Supplementary file 1 [file polymers-14-04637-s001.zip › polymers-1982468-supplementary.pdf]

Supplementary Material: Efficient Lignin Fractionation from Scots Pine (*Pinus Sylvestris*) Using Ammonium-based Protic Ionic Liquid: Process Optimization and Characterization of Recovered Lignin

Sharib Khan, Daniel Rauber, Sabarathinam Shanmugam, Christopher W.M. Kay, Alar Konist and Timo Kikas

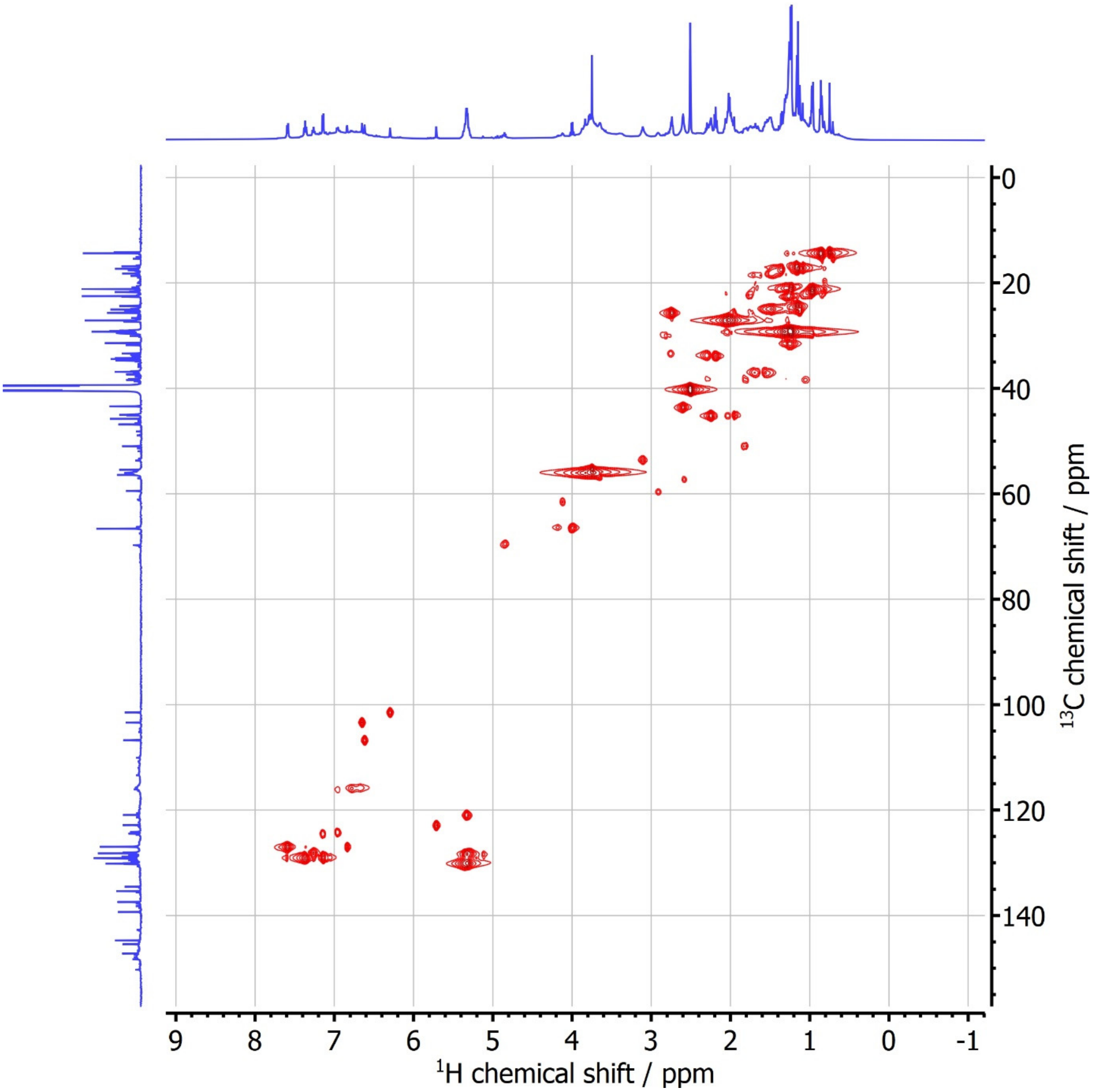

Figure S1. HSQC NMR of [N11H(2OH)][LAC} extracted lignin from pine wood biomass.

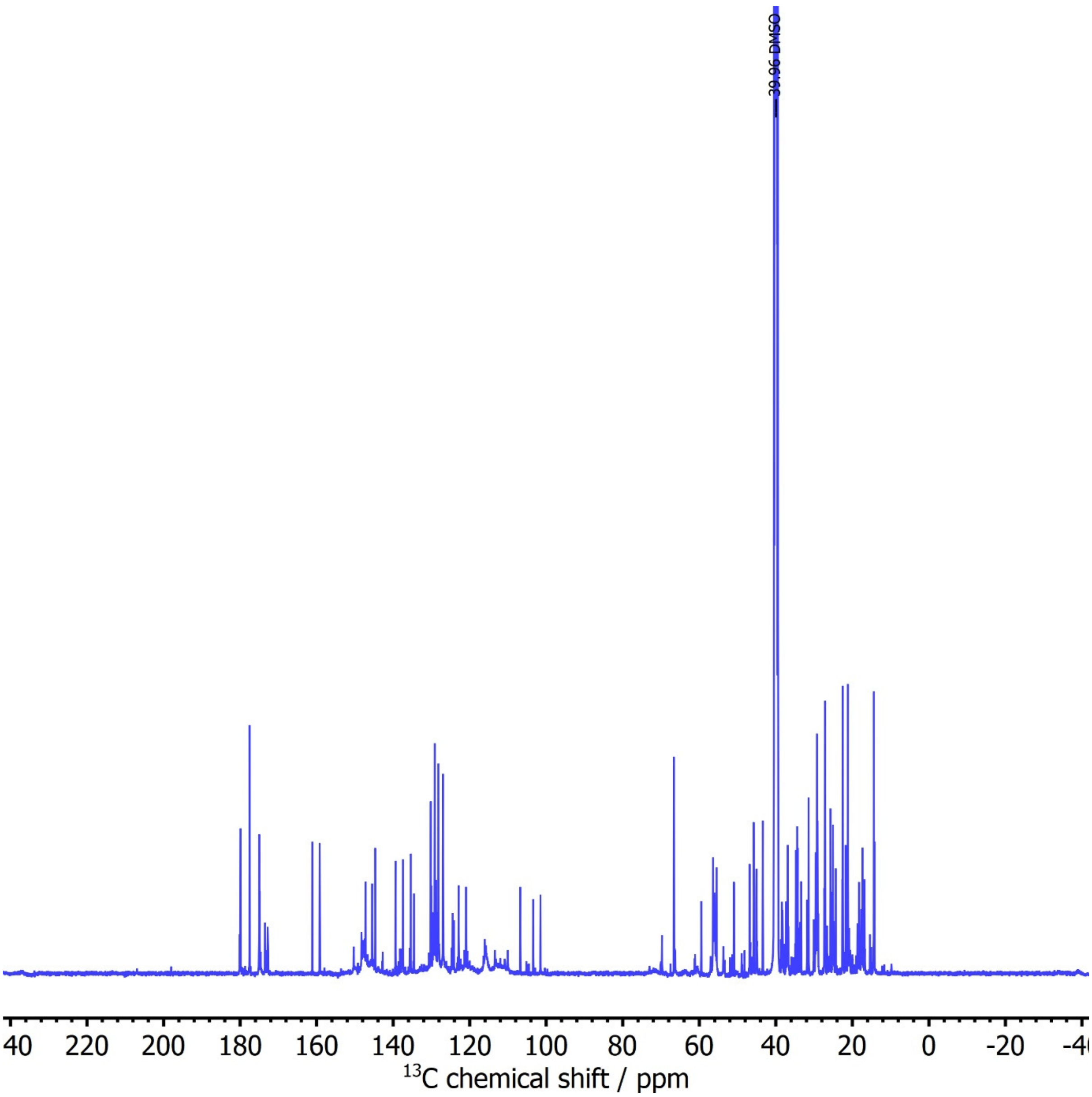

Figure S2. ^{13}C NMR of [N11H(2OH)][LAC] extracted lignin from pine wood biomass.

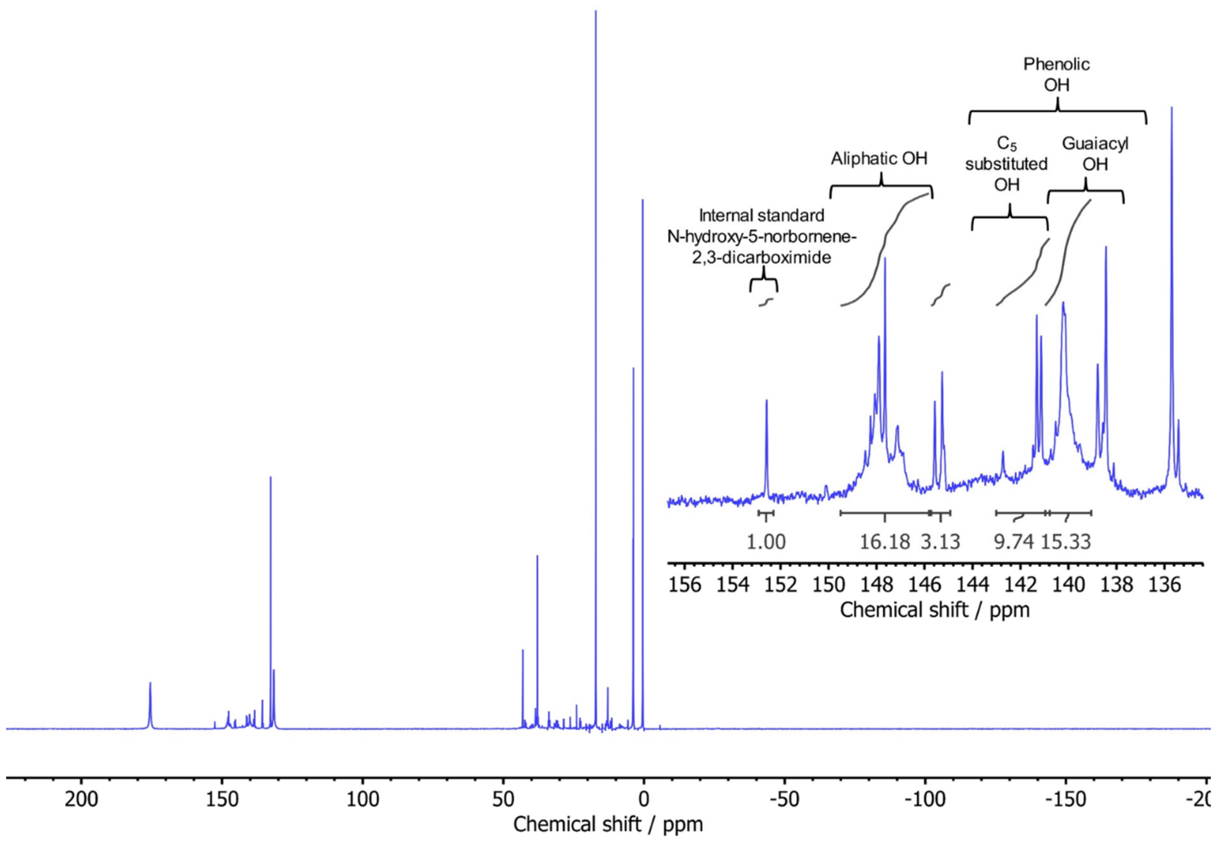

Figure S3. ^{31}P NMR spectrum of the pine wood lignin phosphorylated with 2-chloro-4,4,5,5-tetramethyl-1,3,2-dioxaphospholane (Cl-TMDP).using endo-*N*-hydroxy-5-norbornene-2,3-dicarboximide as internal standard.
